# Global Species Diversity Patterns of Polypodiaceae Under Future Climate Changes

**DOI:** 10.3390/plants14050711

**Published:** 2025-02-26

**Authors:** Sibo Huang, Gangmin Zhang, Wenpan Dong

**Affiliations:** School of Ecology and Nature Conservation, Beijing Forestry University, Beijing100083, China; possible980906@bjfu.edu.cn

**Keywords:** climate change, Polypodiaceae, global warming, species distribution model

## Abstract

Global change influences species diversity patterns. Compared with seed plants, ferns are more sensitive to temperature and humidity changes and are an ideal group for studying species diversity patterns under future climate changes. Polypodiaceae, which has important ecological and application value, such as medicinal and ornamental value, is one of the most widely distributed fern families, with rich species diversity. Here, we explore the changes in the species diversity patterns of Polypodiaceae and their influencing factors. We collected more than 300,000 data points on the distribution of Polypodiaceae to map actual current species diversity patterns. We used Maxent to establish current and future potential species distribution models using 20 predictors and determined the current species diversity patterns using the actual current species diversity patterns and current potential species distribution model method. Multiple linear regression and random forest models were used to evaluate the effects of climate factors on the species diversity patterns of Polypodiaceae. We evaluated the effects of future climate changes on the species diversity of Polypodiaceae. The species diversity of Polypodiaceae increased gradually from higher to lower latitudes and the centers were concentrated in the low latitudes of tropical rainforests. There were four distribution centers across the world for Polypodiaceae: central America, central Africa, southern Asia, and northern Oceania. The species diversity of Polypodiaceae was greatly affected by precipitation factors rather than temperature factors. Under future climate change scenarios, species diversity is expected to shift and accumulate toward the equator in mid-to-low latitudes. Species diversity is projected to remain concentrated in low-latitude regions but will tend to aggregate towards higher altitude areas as global temperatures rise, with precipitation during the warmest season identified as the most influential factor.

## 1. Introduction

Biodiversity is the basis of human survival and the cornerstone of ecological security. It is usually divided into four levels, namely gene, species, ecosystem, and landscape diversity, among which species diversity is the intuitive embodiment of biodiversity. With the continuous intensification of human activities, especially the increased fossil fuel use after the Industrial Revolution, global greenhouse gases have continued to accumulate [1]. Studies have shown that greenhouse gases in the atmosphere will lead to an average increase in global surface temperature of 1.4–5.8 °C by 2100 [2,3]. Climate warming affects the global water cycle process and precipitation distribution patterns [4], further affecting the evolution, extinction, and migration of species [5,6], which changes the combinations and distribution patterns of species in regions and ecosystems throughout the world. Therefore, exploring the diversity pattern and the environmental factors that drive the formation of the pattern is the basis of species diversity conservation.

The impact of climate change on plants varies by taxa, with some species threatened with extinction and others benefiting from range expansions [7,8]. Evaluations of more than 4000 species around the world have shown that about half of their ranges are moving poleward or to higher elevations, but the extent of movement varies by taxa. In Asia, 70% of the species studied have been estimated to be negatively affected by climate change, and about 30% of these species are at a high risk of extinction [9]. Temperature and precipitation are key climate variables that can be used to describe diversity patterns [10]. The relationship between climate and the earth’s communities has been studied for a long time, but until the 1980s and 1990s, ecologists began explicitly examining climate constraints on plants through the lens of physiological ecology [11]. In the context of future climate change, climate datasets and efficient processing systems enable the assessment of its impacts on living organisms and their interactions with the surrounding environment [12]. These are called climate indicators; they affect ecosystems and service bioclimatic indicators (BioClimInd), and there are 19 commonly used biocliminds that describe temperature and rainfall variability, as well as potential changing interactions between the two [13]. Understanding how bioclimatic variables will impact different species and their responses is a hotspot in the biodiversity conservation field. Although species diversity may be influenced by other factors, climate change is one of the most significant drivers of global ecological change, potentially exacerbating these pressures and introducing new challenges. Therefore, understanding future biodiversity patterns under climate change is crucial [14,15,16]. Predicting these patterns effectively requires comprehensive data on current species, which enables the quantification of their conservation status. However, inadequate monitoring often results in missing or incomplete biodiversity data [17]. This lack of comprehensive global distribution data poses significant challenges to studying diversity patterns and hinders progress in designing effective global conservation strategies.

Online databases, such as GBIF (http://www.gbif.org, accessed on 9 May 2023), have been widely used to construct species diversity patterns. However, some data are missing and some of these datasets are far from complete, making complete species mapping at the global scale challenging [18]. The enormous challenge of constructing individual species diversity pattern has been solved with predictions derived from species distribution modellings (SDMs) [19]. Species distribution models (SDMs) are mathematical tools that integrate species presence data with environmental factors to predict habitat suitability. By analyzing statistical information from sampling points, these models estimate the ecological niche requirements of species within a multi-dimensional environmental space. The results are then projected onto a chosen temporal and spatial framework, producing probability maps that reflect the potential suitability of habitats for species. These models provide insights into the distribution of suitable habitats on a large scale, rather than the actual distribution range of species [20].

Over the past 30 years, scientists have developed several models to estimate species distribution and associated environmental variables. Regression algorithms, such as the generalized linear model (GLM), offer a straightforward way to evaluate the influence and importance of environmental variables on species distribution. However, it is relatively difficult to handle large datasets efficiently. In contrast, machine learning algorithms such as random forest (RF) excel at processing vast larger datasets with high accuracy but lack biological interpretability in their modeling processes [20]. The Maxent model, known for its high accuracy with limited sample data, has become a widely used tool for predicting potential suitable habitats, assessing shifts in species distribution, evaluating extinction risks under climate change, conducting biodiversity assessments, and analyzing invasion risks [21,22].

Plants are important ecosystem components, and ferns are a diverse lineage of vascular plants, comprising about 12,000 species [23]. Although the origin of ferns dates back to more than 400 million years ago [24], their large-scale radiation evolution appeared relatively late. The diversity and abundance of living fern species were formed by continuous radiation evolution after angiosperm emergence during the Cretaceous period [25]. Compared with angiosperms, ferns are less efficient at controlling stomatal responses and photosynthesis [1,26,27]. This inefficiency may result in higher evaporative water loss, making ferns highly sensitive to environmental factors such as precipitation and temperature [28]. As a result, fern species diversity serves as an excellent climate indicator [29].

Polypodiaceae, comprising more than 1600 species, has a rich species diversity. This family is widely distributed all over the world, with 89% of species being epiphytic vascular [30]. The number of epiphytic vascular plants accounts for about 10% of global plants [31], but they can account for 50% of local vascular plants in moist forests [32]. Recent studies have shown that the diversity patterns of epiphytic vascular plants differ significantly from those of certain terrestrial representatives, highlighting the importance of investigating the diversity patterns of epiphytic vascular plants under climate change [33]. However, thus far, there has been limited research on the patterns and formation mechanisms of epiphytic plant species diversity [34], especially that of epiphytic ferns. Polypodiaceae consists of six subfamilies: Loxogrammoideae, Platycerioideae, Microsoroideae, Polypodioideae, Grammitidoideae, and Drynarioideae [23]. At present, the establishment of a species distribution database provides a research basis for studying plant diversity patterns.

In this study, we collected more than 300,000 distribution point records for 1052 species covering all distribution areas, mapped the current distribution diversity patterns of Polypodiaceae, and identified distribution hotspots. Based on these data, a species diversity distribution model was projected for different climatic conditions during different periods to reveal the future change trends in species diversity patterns. In addition, multiple linear regression was used to fit the relationship between species richness and climate and environmental factors, such as temperature and precipitation, and the random forest model [35] was used to calculate the weights of different environmental factors, aiming to explore the key climatic factors affecting species diversity patterns. Therefore, the aims of the present study are as follows: (1) To investigate the diversity patterns and hotspot regions within Polypodiaceae species. (2) To reveal the relationship between the diversity patterns and climatic factors. (3) To assess how these patterns may shift under future climate change scenarios.

## 2. Results

### 2.1. Polypodiaceae Species Diversity Patterns

**Figure 1:**
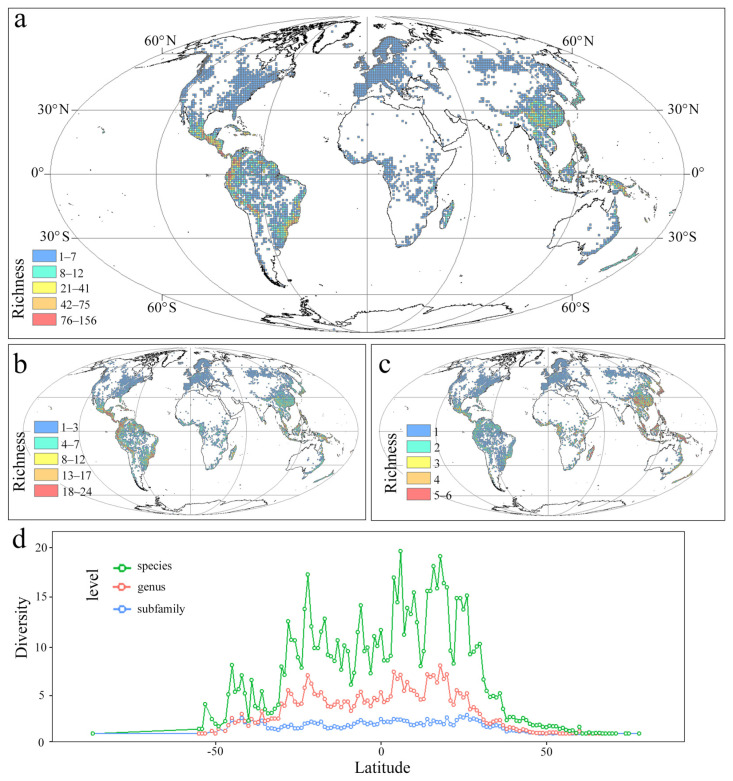
Actual current species diversity patterns of Polypodiaceae. (**a**): Actual current species diversity patterns of Polypodiaceae. (**b**): Actual current genera diversity patterns of Polypodiaceae. (**c**): Actual current subfamily diversity patterns of Polypodiaceae. (**d**): Actual current latitudinal gradient distribution of each subfamily, genus, and species.

In this study, the actual current distribution patterns for species diversity were analyzed based on the distribution data for Polypodiaceae. The species in this family were mainly distributed in tropical rainforest climate zones, subtropical monsoon humid climate zones, and temperate Marine climate zones with high precipitation levels and small temperature differences; they are concentrated in central America, southeast Asia, and northern Oceania. The hotspot distribution zone of the family highly overlapped with tropical rainforests, with the highest diversity grid reaching 156 species (Figure 1a). Except for Antarctica, the species of Polypodiaceae were widely distributed worldwide, and species diversity gradually decreased from low to high latitudes.

**Figure 2:**
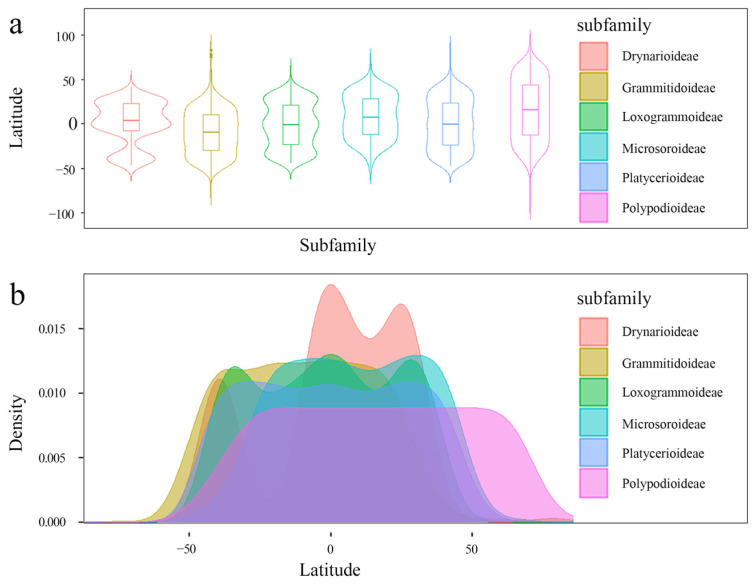
Actual current latitude species diversity patterns of each subfamily. (**a**): Kernel density estimation of the latitude gradient distribution of each subfamily. (**b**): Violin plot of the latitudinal gradient distribution of each subfamily.

**Figure 3:**
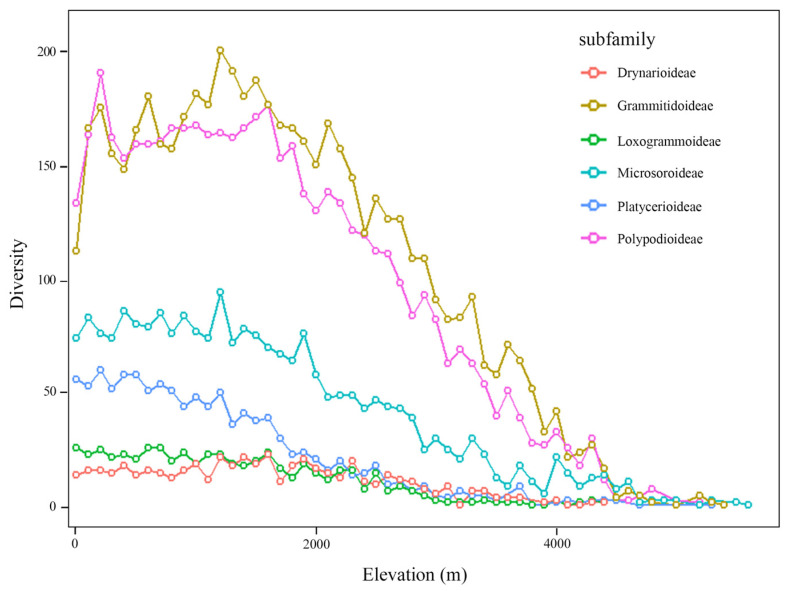
Actual current elevation species diversity patterns of each subfamily.

**Figure 4:**
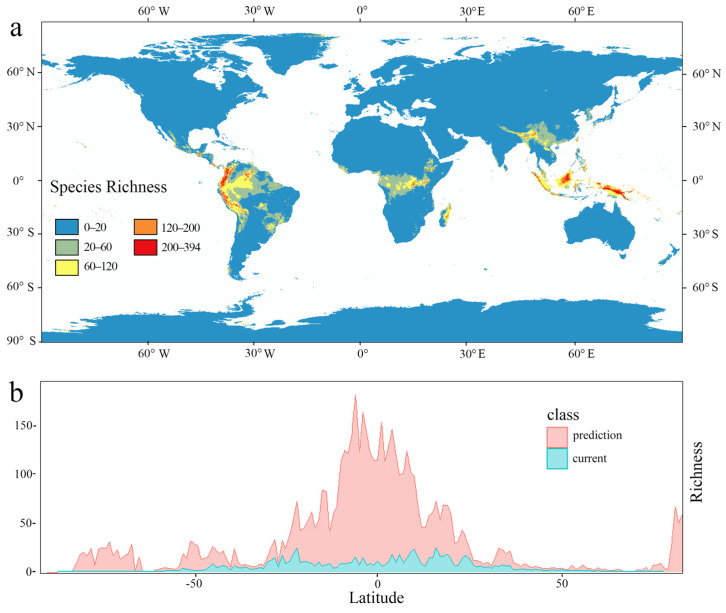
Current potential species diversity patterns of Polypodiaceae using species distribution models. (**a**): Species diversity distribution model from 1991 to 2000. (**b**): Species dimension gradient distribution of Polypodiaceae.

All six subfamilies were actually currently distributed in southeast Asia, which was the hotspot region at the subfamily level. Genera diversity hotspots occurred in the Americas, and the richest grid contained 24 genera (Figure 1). Comparing the three diversity pattern levels in Polypodiaceae, species diversity and genera diversity increased first and then decreased from high to low latitudes. The most abundant species diversity occurred at about 20–30° S and 20–30° N, and Polypodiaceae subfamily diversity was significantly higher from 50° N to 40° S than in other areas (Figure 1d). The kernel density estimation and violin plot showed that all subfamilies except Polypoideae were concentrated between 50° N and 50° S (Figure 2), while the Polypoideae species extended northward to 90° N. Species diversity for each subfamily was concentrated in the low-altitude areas, and it decreased sharply when the altitude was higher than 1700 m (Figure 3). Current potential SDMs showed that species richness ranged from 0 to 394 in a grid cell, and a new species diversity hotspot appeared in central Africa. Comparing each subfamily of SDMs, the species diversity centers were all located in four regions: central America, central Africa, southern Asia, and northern Oceania. The species diversity hotspots based on SDMs were not completely consistent with those based on the distribution records (Figure 4a). Compared with the two species diversity patterns with latitudinal gradients, species diversity both gradually increased from high to low latitudes, but the increasing trend in species diversity was more obvious in current potential SDMs and the species diversity was more abundant (Figure 4b).

The actual current species diversity patterns for subfamilies showed that the species diversity hotspots of Polypodioideae and Grammitidoideae occurred in America, while those of Drynarioideae, Platycerioideae, Loxogrammoideae, and Microsoroideae occurred in southeast Asia and northern Oceania (Figure S1). The highest peak in species diversity within a single grid occurred for 78 species of Polypodioideae, while the lowest peak occurred for the species of Loxogrammoideae. The current potential SDMs showed that each subfamily had four species diversity hotspots at the same time. The highest species diversity was 234 species in Grammitidoideae, and the lowest was 19 species in Loxogrammoideae (Table S1).

### 2.2. Impact of Environmental Variables on Species Diversity Patterns

**Figure 5:**
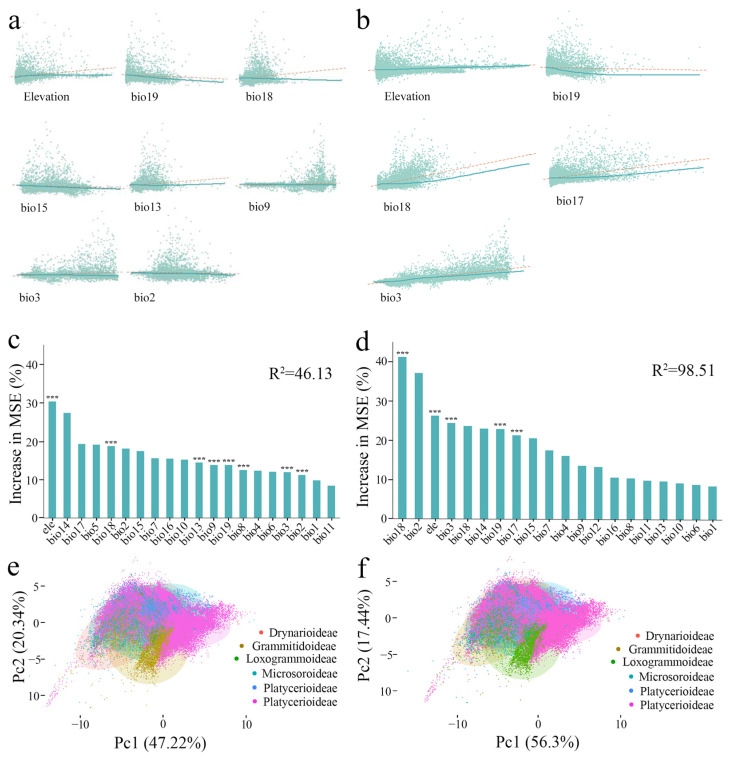
Environmental factors affecting the species distribution pattern in Polypodiaceae. (**a**): Partial residual plot of actual current species diversity and environmental factors (bio19: Precipitation of Coldest Quarter; bio18: Precipitation of Warmest Quarter; bio15: Precipitation Seasonality; bio13: Precipitation of Wettest Month; bio9: Mean Temperature of Driest Quarter; bio3: Isothermality; bio2: Mean Diurnal Range; The red dashed line represents the actual observed values, while the green solid line represents the model’s predicted values or the fitted line.). (**b**): Partial residual plot of species diversity in current potential SDMs and environmental factors from 1991 to 2000 (bio19: Precipitation of Coldest Quarter; bio18: Precipitation of Warmest Quarter; bio17: Precipitation of Driest Quarter; bio3: Isothermality; The red dashed line represents the actual observed values, while the green solid line represents the model’s predicted values or the fitted line.). (**c**): The important values of climate factors in the actual current species diversity of Polypodiaceae. (**d**): Important values of climate factors in the current potential SDMs from 1991 to 2000 (***: correlations between environmental factors and species diversity patterns, *p* < 0.001). (**e**): Principal component analysis of actual current climate effects of subfamilies. (**f**): Principal component analysis of climate effects by subfamilies in the species current potential distribution models.

The relationship between actual current species diversity patterns and 20 environmental factors was analyzed. Species diversity patterns were positively correlated with altitude, Precipitation of the Wettest Month (bio13), and Precipitation of the Warmest Quarter (bio18) and negatively correlated with the diurnal range of Mean Temperature (bio2), precipitation seasonality (bio15), isothermality (bio3), and average temperature of the driest quarter (bio9) (Figure 5). The random forest model was used to obtain a score for the relative importance of each environmental factor. The R^2^ of the simulation results was 46.13. Altitude had the greatest impact on species diversity, with an important value of 30, followed by precipitation of the warmest season (bio18), with an important value of 18. The species diversity and environmental factors for each subfamily were analyzed using principal component analysis. Each subfamily was equally sensitive to the climate, and there was no obvious differentiation in ecological niche.

For current potential SDMs, species diversity was positively correlated with isothermality (bio3), Precipitation of the Wettest Quarter (bio17), Precipitation of the Warmest Quarter (bio18), and altitude and significantly negatively correlated with Precipitation of the Coldest Quarter (bio19). Using the random forest model, the R^2^ of the simulation results was 98.51. Precipitation of the warmest season (bio18) had the greatest impact on the correlation of species diversity, with an important value of 41.21, followed by altitude, with an important value of 26.25 (Table 1). This demonstrates that precipitation-related factors have a great impact on species diversity. Principal component analysis for each subfamily and environmental factor in the SDMs from 1991 to 2000 showed no obvious niche differentiation among subfamilies (Figure 5e,f).

### 2.3. Future Species Distribution Model of Polypodiaceae

**Figure 6:**
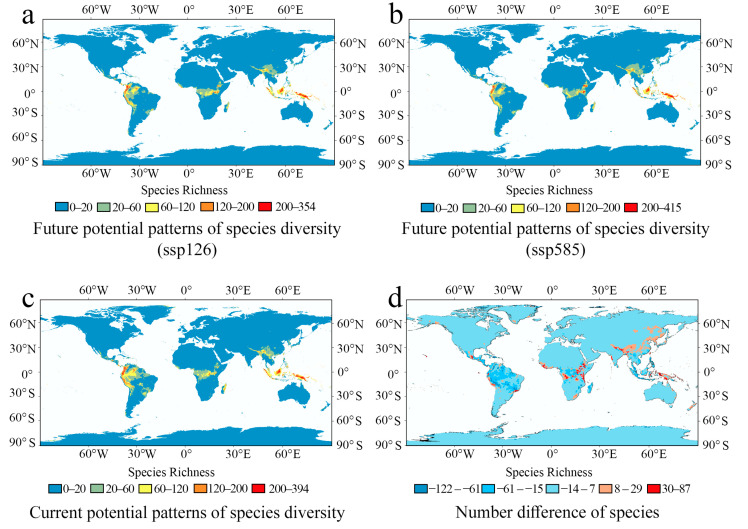
Future potential species diversity pattern changes in Polypodiaceae under climate change. (**a**): Potential patterns of species diversity under future low-carbon-dioxide-concentration scenarios (ssp126). (**b**): Potential patterns of species diversity under future high-carbon-dioxide-concentration scenarios (ssp585). (**c**): Current potential patterns of species diversity. (**d**): The change in species diversity of Polypodiaceae over the next 100 years (potential patterns of species diversity under future high-carbon-dioxide-concentration scenarios minus current potential patterns of species diversity).

Future potential SDMs of 20 environmental factors under two future climate scenarios (period 2081–2100) were constructed and compared with the current potential SDMs (Figure 6). After subtracting the current potential SDMs from the future potential SDMs under high CO_2_ emission pressure (period 2081–2000), we obtained a diversity difference value for Polypodiaceae (Figure 6d). The future potential patterns of species diversity remain unchanged, with four distinct patterns of species diversity persisting in low-latitude regions, and the peak values show little variation across different CO_2_ emission scenarios in various models. Although the future potential species richness does not decrease, the patterns of species diversity tend to become more concentrated and shift towards higher altitude areas; specifically, species diversity increases in the Himalayan and Andes mountains ranges, while it decreases in the Amazon Basin and the Congo Basin.

## 3. Discussion

In this study, the multi-layer overlay method was used to discover the current potential species diversity patterns of Polypodiaceae and to assess its variation in the future under climate change. Actual current species diversity was concentrated in central America, southeast Asia, and northern Oceania, while the current potential SDMs showed that the species diversity was concentrated in central America, central Africa, southeast Asia, and northern Oceania. In general, the hotspots and maximum diversity in potential SDMs for Polypodiaceae will change little on a global scale in the future (2081–2100) compared with the present situation. Species diversity in middle and low latitudes will shift toward the equator and have four biodiversity hotspots. In this study, relevant variables such as elevation, temperature, and precipitation were used to analyze the driving factors of species diversity patterns. In contrast to the species diversity patterns of Polypodiaceae observed in IPCC (2014) [36], this study showed that they were more driven by precipitation.

### 3.1. Species Diversity Hotspots of Polypodiaceae

Epiphytic plant species account for as much as 25% in some tropical rainforests and are an important component of local species diversity [37]. There are three hotspots of the actual current Polypodiaceae species diversity: central America, southeast Asia, and northern Oceania. All of these concentrate at low latitudes, and most areas are tropical rainforests, similar to the fern species diversity map drawn by Qian et al. [38]. The tropical rainforest is not only a refuge for organisms but also an important water source. High-latitude tropical rainforests (tropical cloud forests) are the cradle of Polypodiaceae. Tropical cloud forests are about 1700 m above sea level, with low temperatures and high humidity, which are suitable conditions for epiphytic plant growth. This is well illustrated in the actual current elevation gradient map of species diversity, which concentrates at an altitude of 1700 m (Figure 3). This is similar to the result of Hernández-Rojas et al. [39].

Compared with the overall species diversity pattern of epiphytic vascular plants, the actual current species diversity of Polypodiaceae in Africa is noticeably lower. Apart from potential sampling biases that may lead to missing data, this phenomenon may be related to the origin and dispersal of Polypodiaceae. This family diverged from the paleotropics at 55 Ma, then migrated to the neotropics at 43 Ma, and diversified rapidly [40]. The dispersal to Africa was relatively late, and there was little differentiation in the diversity of species in Africa [30,40]. At the end of the Oligocene, a single lineage, which constituted half of Grammitidoideae, migrated to tropical Asia instead of Africa. This migration process continued until the Miocene [40]. It is well known that the dry conditions of the Miocene led to mass extinction, and that alternating dry and wet conditions in Africa during this period allowed some species to diverge rapidly at certain refuge time points [41,42]. However, through the study of Testo [24], we found that many species of Polypodiaceae were in a stage of rapid radiation evolution and differentiation during this period, which continues to the present, and did not change the rate of diversification during this period. So, we think that Polypodiaceae did not spread to Africa during the Pleistocene and quickly became extinct. This result suggests that the origin and spread of taxa also play crucial roles in the current species diversity patterns [43].

Suissa et al. [44] used GBIF data to draw a 100 × 100 km actual current pattern of species diversity, but GBIF data were incomplete or uneven [45], and the current potential SDMs we established made up for this defect through the algorithm to simulate the potential species distribution. In addition, since the SDM result is a potential distribution pattern simulated according to the correspondence between bioclimatic factors and species distribution, the species diversity in the simulated result is much higher than the existing species diversity, which is normal. This is precisely because SDM is a supplement to the incomplete sampling data, especially the Grammitidoideae in the Department of Polypodiaceae. Weigand et al. [46] mapped the SDMs of ferns using a generalized linear model. They obtained 99 observations in the unit quadrat and 439 simulations. SDMs supplemented the distribution data, which is conducive to the subsequent analysis of species diversity patterns and environmental factors. For each subfamily, there were always four centers of species diversity in the SDMs, while the current subfamily species had different centers. This may be due to incomplete sampling or late spread of Polypodiaceae into Africa, rather than an inappropriate environment. There are many tropical rainforests distributed in the species diversity centers, coinciding with the distribution patterns of epiphytes and indicating that these centers should have been suitable for the survival of Polypodiaceae. The species diversity in the simulated results was much higher than the current species diversity, which may be related to the incomplete sampling of species distribution data, especially that for Grammitidoideae (Figure S2).

### 3.2. Effects of Different Environmental Factors on Species Diversity Patterns

The impact of environmental factors on species diversity patterns has been analyzed in previous studies, including temperature, precipitation, soil, elevation, and slope [47]. Because of their unique structures, different plant groups have varying responses to bioclimate factors. It is very important to determine which factors affecting species diversity patterns are dominant. Global climatic conditions can lead to the aggregation of specific plant groups, while niche differentiation may be the main cause of species diversification [48]. At present, biological and environmental factors are considered the two major factors affecting species diversity, among which temperature and precipitation are the most important [49]. The altitude distribution of ferns is determined by a wide range of factors, not only due to climate factors but also other unknown potential factors. Therefore, the correlation between species diversity and altitude is not excluded after calculating the autocorrelation of various climate factors in this paper [50]. This study mainly analyzed the influence of temperature, precipitation, and other related factors on species diversity patterns. Multiple linear regression and random forest model comparison analyses showed that the species diversity patterns of Polypodiaceae were more coupled with climatic factors related to precipitation than temperature, consistent with a previous study [33]. Qian assessed the relationship between metrics of species and phylogenetic structure and six climatic variables [51]. They think that when old clades and polypods were considered separately, temperature-related variables explained more variation in fern diversity than did precipitation-related variables. However, after we used all the climatic variables to calculate the VIF to assess the autocorrelation between factors, we found that the VIF values of the six climatic variables mentioned by Qian were all greater than 10. This indicates that these six climatic variables exhibit autocorrelation with other climatic variables and therefore need to be deleted. Compared with other taxa, the epiphytic groups accounted for a relatively larger proportion of Polypodiaceae, which was conductive to water capture, storage, and utilization. Future changes in the diversity patterns of Polypodiaceae are more likely to be affected by precipitation, differing from those of other plants migrating to higher latitudes.

Current potential SDMs are more strongly correlated with precipitation than actual current species diversity patterns. The actual current species diversity patterns of Polypodiaceae are not only restricted by the climatic environment but are also influenced by the origin and spread. The simulated analyses were supplemented by missing data from the sample, which were more sensitive to environmental factors and could more intuitively reflect the changing group diversity patterns.

Principal component analysis was conducted on the actual current and current potential species richness and environmental factors of each subfamily. There was no obvious differentiation in the ecological niche, indicating that the distribution patterns should be the same for several subfamilies. The differences in distribution patterns may be due to different results based on the different migration and evolutionary histories of each subfamily or sampling loss from GBIF. Regarding future research, there are still no differentiation trends for the ecological niche of each subfamily.

In addition to the origin and spread history, current species diversity patterns have been greatly influenced by climate factors, such as precipitation in the warmest quarter. With the gradual warming of the global climate in the future, it is important to simulate the future optimal distribution. This can intuitively show the future change trends in species hotspots with temperature and precipitation.

### 3.3. Future Potential Distribution Variation in Polypodiaceae Under Climate Change

By comparing the three models with different CO_2_ emission scenarios, the species diversity of Polypodiaceae remains concentrated in low-latitude regions, and the patterns of species diversity have not changed, but they have become more concentrated. This is inconsistent with conclusions in other taxa [52]. Liang et al. [53] found that rice migrated to high latitudes from 1984 to 2013, differing from the species diversity of Polypodiaceae. Compared with other taxa, Polypodiaceae contains a large proportion of epiphytic taxa, which have a high demand for water. With global warming in the future, precipitation will increase near the equator, resulting in the aggregation of Polypodiaceae species in this area. However, the shift in Polypodiaceae species diversity towards higher altitudes aligns with the findings of He’s research [54]. Unfortunately, at present, we have not found a suitable method to apply phylogenetic results to SDMs, so in the follow-up study, we hope to find a suitable method to establish a prediction model of phylogenetic diversity of Polypodiaceae.

## 4. Materials and Methods

### 4.1. Species and Distribution Records

In this study, Polypodiaceae taxonomy followed PPG I [23]. We used the accepted genus and the number of species within each genus from the PPG I system as a benchmark and relied on a series of lists. The list of species of Polypodiaceae we collected, comprising 1592 species covering 65 genera in six subfamilies, was retrieved from Plants of the World Online (https://powo.science.kew.org, accessed on 9 May 2023), the Plant List (http://www.theplantlist.org, accessed on 9 May 2023), world plants (https://www.worldplants.de/world-plants-complete-list/complete-plant-list, accessed on 9 May 2023), and World Flora Online (https://www.worldfloraonline.org, accessed on 9 May 2023). To construct the current species diversity patterns, more than 1 million distribution datapoints were provided by GBIF (https://www.gbif.org/, accessed on 9 May 2023) [55] and CVH (https://www.cvh.ac.cn/, accessed on 9 May 2023). Due to the presence of many homotypic and heterotypic synonyms in GBIF, we filtered the species data from GBIF according to our species list. After excluding some species without distribution information, we obtained more than 679,976 datapoints for 1052 species all over the world. Arcgis10.8 was used to delete erroneous data and duplicate records. We removed records with (0, 0) coordinates, coordinates located in the ocean, duplicate biodiversity records within 1 km of each other, and records with reversed latitude and longitude values. Then, R package ‘rbokeh’ was used to generate maps at the genus level of species occurrences to identify further erroneous records; species outside of their native range were removed. A total of 337,695 distribution datapoints were obtained for Polypodiaceae.

### 4.2. Environmental Data

Previous studies have shown that climatic variables are suitable predictors of species richness for ferns [45]. All climatic variables were used in this study. Nineteen bioclimatic and elevation data characteristics were obtained from WorldClim 2.1 (https://worldclim.org/, accessed on 9 May 2023) [56], with a spatial resolution of 2.5 arc-minutes. Bio1–Bio10 were temperature-related climate factors, and Bio11–Bio19 were precipitation-related climate factors (Table 1). Among them, the CMCC-ESM2 atmospheric circulation model for future climate selection was derived from two shared socioeconomic path scenarios (ssp126 and ssp585) [57], with ssp126 representing the scenario of lower greenhouse emissions and ssp585 representing the scenario of higher greenhouse emissions. CMCC-ESM2 is one of the participating models in CMIP6 (the sixth phase of the Coupled Model Intercomparison Project). As a fully coupled Earth system model, it is capable of simulating interactions among the atmosphere, ocean, land surface, cryosphere, and biogeochemical processes. Its simulation results show a high degree of consistency with observational data, and the model has gained recognition from the international scientific community [58]. Due to potential correlations among certain environmental variables, there is a risk of overfitting in the model analysis results. To address this issue, we conducted screening and correlation analysis on the environmental variables. Initially, all environmental variables were preliminarily simulated in the model to calculate the species diversity patterns. Subsequently, stepwise regression and Variance Inflation Factor (VIF) analysis were employed to eliminate the autocorrelation among environmental factors. The VIF, defined as the ratio of the variance in the presence of multicollinearity among independent variables to the variance in its absence, serves as an indicator of collinearity severity and is the reciprocal of tolerance. A higher VIF value denotes a more pronounced collinearity issue. Specifically, a VIF exceeding 10 is indicative of a robust collinear relationship [59]. We removed environmental factors with VIF values greater than 10 and those unrelated to species diversity, then re-ran the simulation.

### 4.3. Actual Current Species Diversity Patterns of Polypodiaceae

Species richness was analyzed at the species, genus, and subfamily levels using grid cell sizes of 100 km × 100 km. “Create Fishnet” in ArcMap10.8 was used to generate grid cells based on the world map, and the sorted geographical distribution data were projected into the grid. The number of species in each network was calculated, and the species richness of different grids was displayed with the help of layer attributes. Using a 100 km × 100 km grid cell size resulted in 25,115 grid cells across the world with at least one Polypodiaceae record. When the data were aggregated within the grid cells, the 338,313 records were reduced to 35,882 unique occurrences.

We created line charts in R to visualize richness gradients for Polypodiaceae in a 1° resolution over latitudinal grid space using diversity pattern data. Moreover, we created a kernel density estimation and a violin plot of each subfamily in R 4.4.2.

### 4.4. Modeling of Species Distribution Models (SDMs)

MaxEnt used information entropy principles to simulate the potential distribution of target species based on the actual species distribution sites and the environmental variables in the species distribution area. Compared with other SDMs, its predictive ability is more reliable on incomplete information and sampling [22]. Maxent3.2.2 [60] was selected to simulate species distribution using 20 climate and environmental factors (Figure S3). Distribution data for 1052 species were simulated under two climatic conditions: present (1991–2000) and future (2081–2100, ssp126, and 2081–2100, ssp585). For species with 30 or fewer distribution points, we employed the bootstrap method; for those with more than 30 distribution points, we utilized cross-validation for the calculations. Data simulation was adopted with a 75% training set, a 25% test set, and 10 repetitions for each species.

The receiver operating characteristic (ROC) curve was used as a measure of the model prediction effect. The area under the curve (AUC) value is the area under the ROC curve ranging from zero to one; the values closer to one indicate that the model performs better than a random model (measuring the accuracy of diagnostic systems). The evaluation criteria were as follows: poor [0–0.7], good [0.7–0.9], excellent [0.9–1]. In our study, models with an AUC greater than 0.8 were selected. We did not carry out mask extraction on the data because of the global distribution of Polypodiaceae. The background points were set to the software’s default of 10,000 [61].

To convert the suitability values of species potential distribution models for individual species into a threshold for presence/absence data, Arcgis10.8 was used to project the results of models onto the Mollweide coordinate system and to reclassify each model, and the predicted results were specified as two ranges, classifying values 0–0.5 as 0, indicating absence, and values 0.6–1 as 1, indicating presence. The SDMs under each climatic condition were superimposed to generate prediction maps of species diversity patterns. The latitudes for species diversity were drawn using R.

### 4.5. Relationship Between Bioclimatic Factors and Species Diversity

To explore the underlying driving forces of Polypodiaceae diversity patterns, all 20 environmental variables were extracted as mean values and species diversity was extracted as maximum values within each 100 km × 100 km grid cell using the “Zonal Statistic as Table” function in the Arc Toolbox from ArcMap10.8 software. A data matrix was formed to resolve the grid number and all environmental factors.

Although the model can calculate the relationship between each species and environmental factors, it cannot determine the relationship between species diversity and environmental factors. To address this, we employed a random forest model to calculate the relative importance scores of various environmental factors [17]. Additionally, we conducted multiple regression analysis on the actual current species diversity patterns and used the random forest model to compute the weights of environmental factors. This allowed us to compare the differences in the correlations between environmental factors and both the actual current species diversity and the current potential species diversity. Multiple linear regression was performed using the “ggplot2” package in R. The “car” package in R was used to draw the partial residual plot, and its linear relationship and significance were determined. The “randomForest” package in R was used to simulate the random forest model. The weights of the different climate and environmental factors were calculated. We aimed to use a random forest model to fit the relationship between species abundance and environmental factors to determine which factors have a greater impact on species diversity. In the results, the R-squared value reflects the overall explanatory power of the predictor variables for the response variable, indicating a high goodness of fit of the random forest model. The “%IncMSE” (increase in mean squared error) measures variable importance by randomly permuting each predictor variable—if a variable is more important, replacing its values randomly will result in a greater increase in model prediction error. A higher value indicates greater importance of that variable.

## Figures and Tables

**Table 1:** Interpretation of degree and significance of environmental factors for species diversity.

		Actual Current			Current Potential		
	% Var Explained	46.13			98.51		
Code	Environmental Factor	%IncMSE	IncNodePurity	*p*	%IncMSE	IncNodePurity	*p*
bio19	Precipitation of Coldest Quarter (mm)	13.77	24,652.16	***	22.91	137,277.80	***
bio18	Precipitation of Warmest Quarter (mm)	18.69	56,010.16	***	41.21	1,859,578.50	***
bio17	Precipitation of Driest Quarter (mm)	19.22	29,175.40		21.39	753,701.80	***
bio16	Precipitation of Wettest Quarter (mm)	15.45	55,450.79		10.72	1,751,408.30	
bio15	Precipitation Seasonality (mm)	17.39	23,001.16		20.62	212,984.20	
bio14	Precipitation of Driest Month (mm)	27.33	36,040.37		23.00	641,513.60	
bio13	Precipitation of Wettest Month (mm)	14.43	49,049.15	***	9.79	1,265,068.90	
bio12	Annual Precipitation (mm)	11.18	25,203.64		13.34	3,100,884.00	
bio11	Mean Temperature of Coldest Quarter (°C)	8.42	39,532.36		10.00	1,076,068.20	
bio10	Mean Temperature of Warmest Quarter (°C)	15.11	46,142.62		9.32	385,357.20	
bio9	Mean Temperature of Driest Quarter (°C)	13.78	28,886.71	***	13.64	647,472.90	
bio8	Mean Temperature of Wettest Quarter (°C)	12.42	27,365.41	***	10.54	295,525.30	
bio7	Temperature Annual Range (°C)	15.52	28,086.18		17.58	6,507,576.70	
bio6	Min Temperature of Coldest Month (°C)	12.02	42,866.75		8.93	1,050,860.80	
bio5	Max Temperature of Warmest Month (°C)	19.07	27,919.79		23.70	1,362,979.10	
bio4	Temperature Seasonality	12.25	42,129.15		16.17	3,668,134.30	
bio3	Isothermality	11.87	44,677.82	***	24.43	2,608,816.50	***
bio2	Mean Diurnal Range (Mean of Monthly) (°C)	17.98	26,824.56	***	37.13	430,218.70	
bio1	Annual Mean Temperature (°C)	9.78	41,146.20		8.53	493,753.80	
elevation	Elevation	30.24	83,199.00	***	26.25	914,869.30	***

***: *p* < 0.001, indicating that the result is significant at the 0.1% significance level.

## Data Availability

Our data can be shared after the article is accepted. The data used for this study are available to download from Dryad. (Reviewer URL: http://datadryad.org/stash/share/F8pI7MWB4jR5FzabqIoPm3k3m-8uoA_3TGduuJn8Ock, accessed on 9 May 2023).

## Supplementary Materials

The following supporting information can be downloaded at https://www.mdpi.com/article/10.3390/plants14050711/s1. Figure S1: Actual current species diversity patterns of subfamilies; Figure S2: Current potential species diversity patterns of subfamilies; Table S1: The range of species diversity in actual current species diversity patterns and current potential; Figure S3: Flowchart of the modeling process of the species distribution models used in this study, including data collection, data cleaning, and map and overlay construction; Figure S4: Species diversity differences among subfamilies; Table S2: The environmental data and their VIF value after removing autocorrelation using VIF values.
