# Supplementary material for: Global Species Diversity Patterns of Polypodiaceae Under Future Climate Changes
Source: Plants (Basel). 2025 Feb 26;14(5):711. doi: 10.3390/plants14050711 (PMC11902136; doi:10.3390/plants14050711)
Supplement: Supplementary file 1 [file plants-14-00711-s001.zip › plants-3457869-supplementary.pdf]

Supplementary Materials

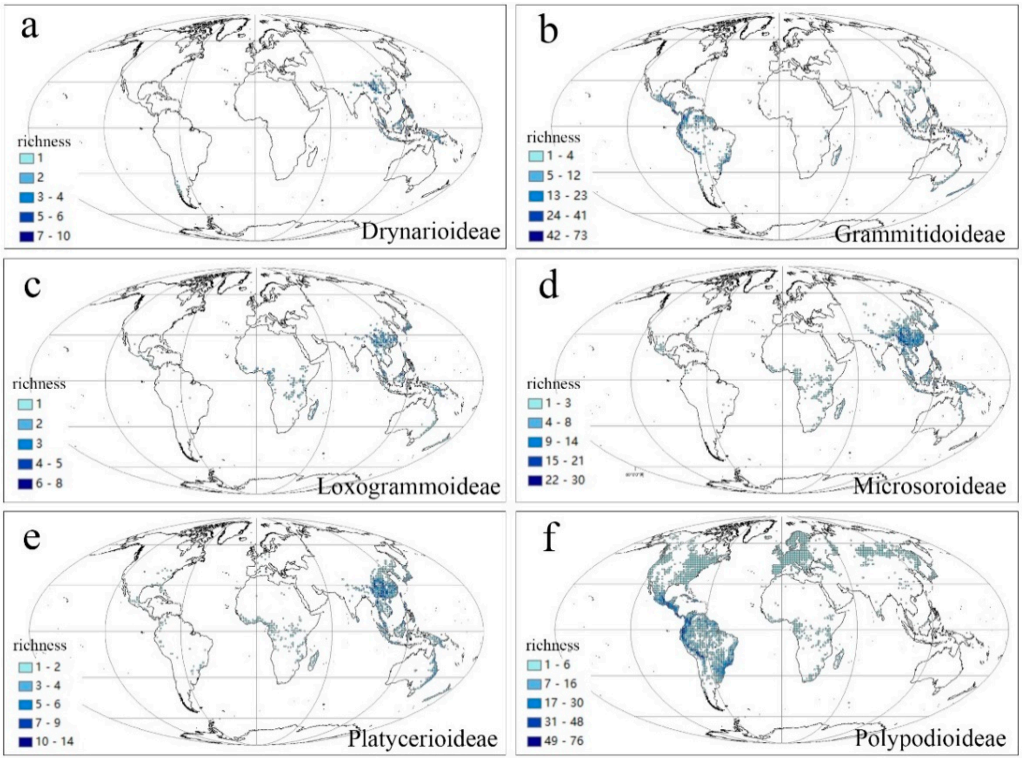

FIgure S1 Actual current species diversity patterns of subfamilies.

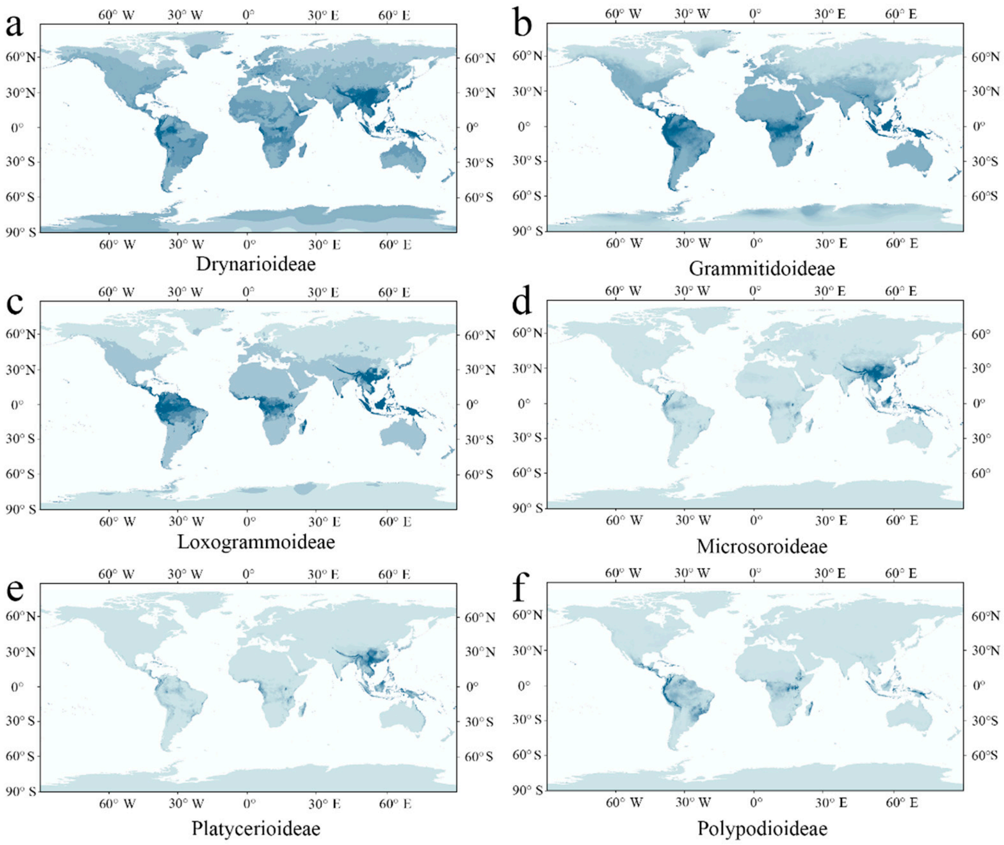

FIgure S2 Current potential species diversity patterns of subfamilies.

Table S1 The range of species diversity in actual current species diversity pattern and current potential.

Actual current species diversity pattern		Current potential SDMs from 1991 to 2000	
The range of diversity	0-156	The range of diversity	0-596
subfamily	The range of diversity	subfamily	The range of diversity
Drynarioideae	0-10	Drynarioideae	0-21
Grammitidoideae	0-75	Grammitidoideae	0-234
Microsoroideae	0-31	Microsoroideae	0-48
Platycerioideae	0-18	Platycerioideae	0-29
Polypodioideae	0-78	Polypodioideae	0-106
Loxogrammoideae	0-8	Loxogrammoideae	0-19

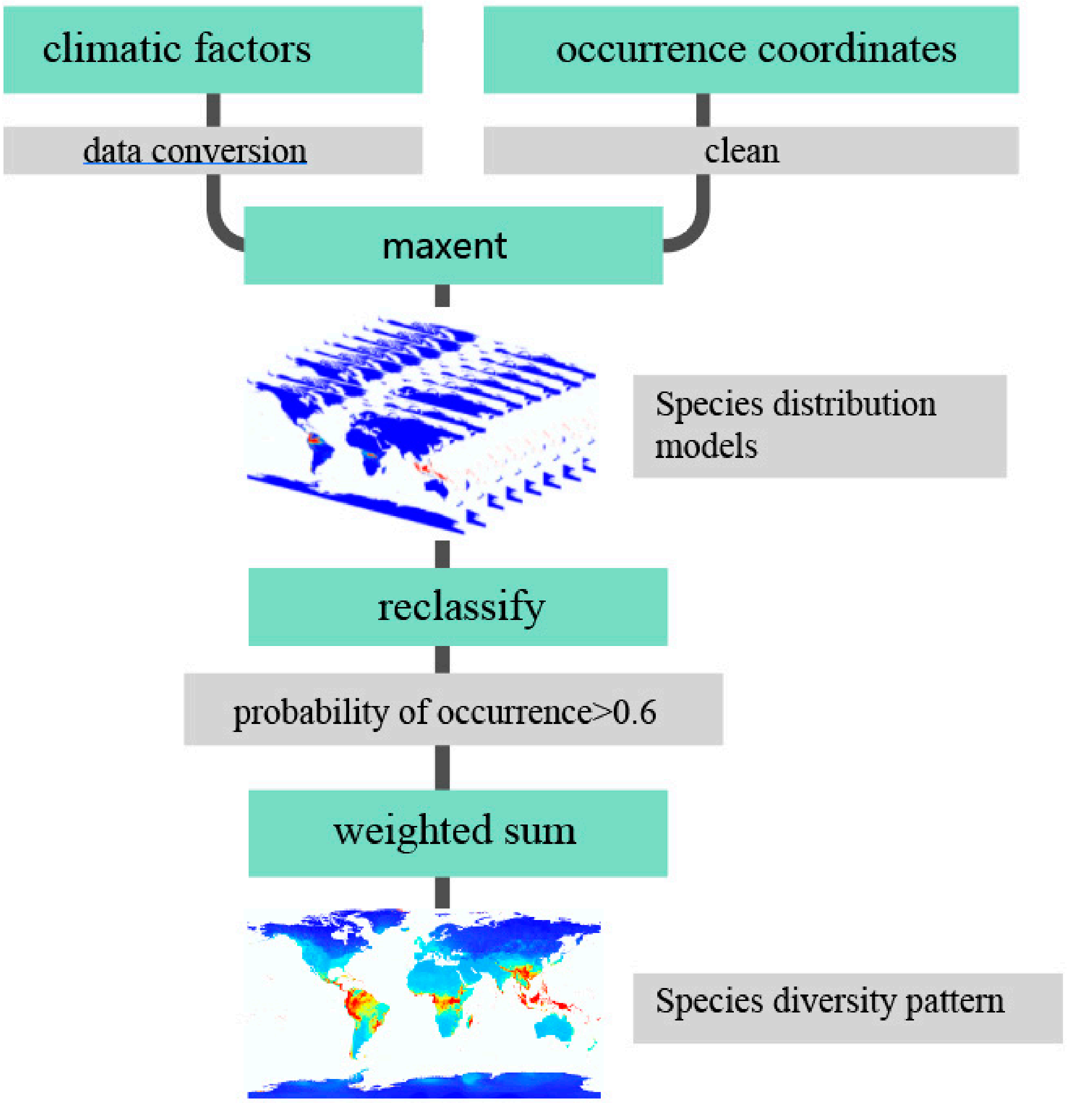

FIgure S3 Flowchart of the modeling process of the species distribution models used in this study, including data collection, data cleaning, and map and overlay construction.

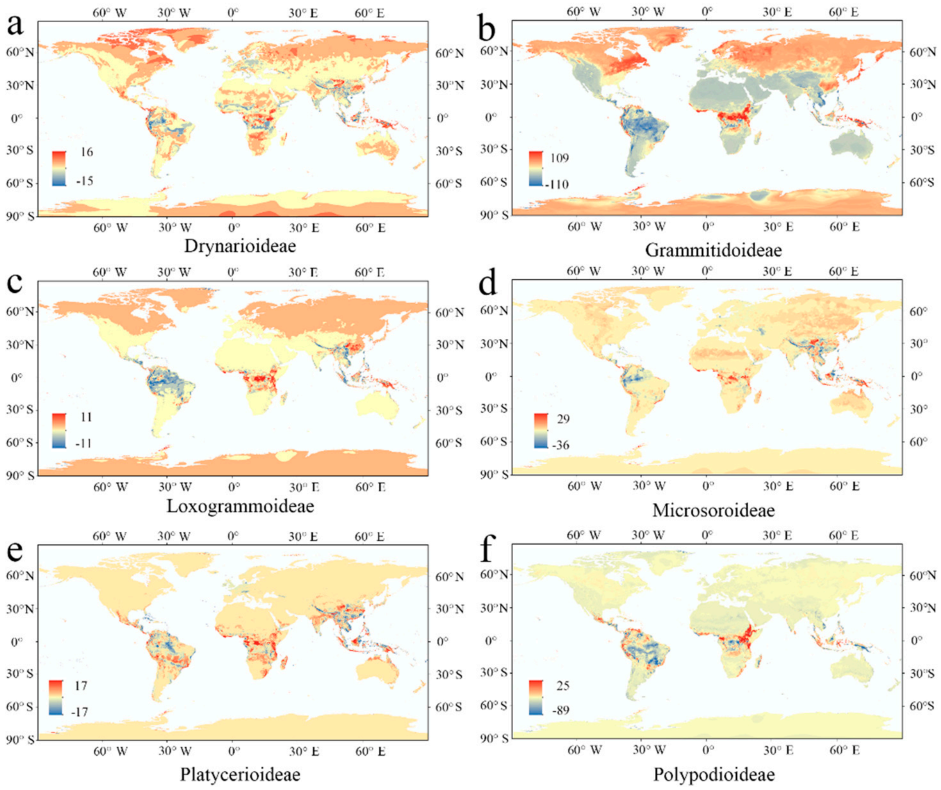

FIgure S4 Species diversity differences among subfamilies.

Table s2 After removing autocorrelation using VIF values, the environmental data and their VIF value.

	VIF
ele	4.420572
bio19	5.157400
bio18	7.829648
bio17	4.822338
bio3	9.096727
